# A Virtual Reality Game Suite for Graded Rehabilitation in Patients With Low Back Pain and a High Fear of Movement: Within-Subject Comparative Study

**DOI:** 10.2196/32027

**Published:** 2022-03-23

**Authors:** Alexander T Peebles, Susanne van der Veen, Alexander Stamenkovic, Christopher R France, Peter E Pidcoe, James S Thomas

**Affiliations:** 1 Department of Physical Therapy Virginia Commonwealth University Richmond, VA United States; 2 Department of Engineering Laurel Ridge Community College Warrenton, VA United States; 3 College of Arts and Sciences Ohio University Athens, OH United States

**Keywords:** virtual reality, reaching, intervention, rehabilitation, exergaming, biomechanics, serious games, gamification, movement, physiotherapy, lumbar

## Abstract

**Background:**

Complex movement pathologies that are biopsychosocial in nature (eg, back pain) require a multidimensional approach for effective treatment. Virtual reality is a promising tool for rehabilitation, where therapeutic interventions can be gamified to promote and train specific movement behaviors while increasing enjoyment, engagement, and retention. We have previously created virtual reality–based tools to assess and promote lumbar excursion during reaching and functional gameplay tasks by manipulating the position of static and dynamic contact targets. Based on the framework of graded exposure rehabilitation, we have created a new virtual reality therapy aimed to alter movement speed while retaining the movement-promoting features of our other developments.

**Objective:**

This study aims to compare lumbar flexion excursion and velocity across our previous and newly developed virtual reality tools in a healthy control cohort.

**Methods:**

A total of 31 healthy participants (16 males, 15 females) took part in 3 gamified virtual reality therapies (ie, Reachality, Fishality, and Dodgeality), while whole-body 3D kinematics were collected at 100 Hz using a 14-camera motion capture system. Lumbar excursion, lumbar flexion velocity, and actual target impact location in the anterior and vertical direction were compared across each virtual reality task and between the 4 anthropometrically defined intended target impact locations using separate 2-way repeated measures analysis of variance models.

**Results:**

There was an interaction between game and impact height for each outcome (all P<.001). Post-hoc simple effects models revealed that lumbar excursion was reduced during Reachality and Fishality relative to that during Dodgeality for the 2 higher impact heights but was greater during Reachality than during Fishality and Dodgeality for the lowest impact height. Peak lumbar flexion velocity was greater during Dodgeality than during Fishality and Reachality across heights. Actual target impact locations during Dodgeality and Fishality were lower relative to those during Reachality at higher intended impact locations but higher at lower intended impact locations. Finally, actual target impact location was further in the anterior direction for Reachality compared to that for Fishality and for Fishality relative to that for Dodgeality.

**Conclusions:**

Lumbar flexion velocity was reduced during Fishality relative to that during Dodgeality and resembled velocity demands more similar to those for a self-paced reaching task (ie, Reachality). Additionally, lumbar motion and target impact location during Fishality were more similar to those during Reachality than to those during Dodgeality, which suggests that this new virtual reality game is an effective tool for shaping movement. These findings are encouraging for future research aimed at developing an individualized and graded virtual reality intervention for patients with low back pain and a high fear of movement.

## Introduction

Virtual reality (VR) has emerged as a promising tool for psychological and movement-based rehabilitation. For example, VR has been used to improve gait adaptability and stability in populations with mobility impairment and a heightened risk for falls [1,2], alleviate phantom limb pain in patients with upper extremity amputation [3], reduce combat-related posttraumatic stress in active duty service members [4], and improve proprioception, mobility, and muscle strength in older adults with knee osteoarthritis [5]. A clear advantage of the VR environment is that it can provide a gamified intervention that is designed to increase enjoyment, motivation, and retention. This can be particularly beneficial when the goal of the intervention is to stimulate movements that may be associated with pain and fear. Based on these unique advantages and the increase in user-friendly VR systems that continue to reduce in cost, it is likely that VR could become a fundamental component of psychological and movement-based rehabilitation programs.

Our group has been developing and testing novel VR games to assess and improve movement deficits in patients with low back pain (LBP) [6]. LBP is the most common source of pain among middle-aged and older adults, resulting in significant financial impact through both health care costs and pain-related work absences [7,8]. Much of these health care costs are driven by the approximately 10% of patients who develop chronic LBP that lasts for many years [9]. Many patients with LBP will develop kinesiophobia, which is fear of movement due to expectations of pain and harm [10]. Among patients with LBP, kinesiophobia is associated with reduced physical activity and particularly reduced spine motion and is a strong predictor of chronic LBP development [10-14]. In brief, kinesiophobia is central to the development of chronic pain through a vicious cycle of pain catastrophizing, hypervigilance regarding pain-related threat, fear-related avoidance of movement, and a resultant combination of disuse, physical deconditioning, and depression, which serve to further amplify pain [10,11,13].

**Figure 1:**
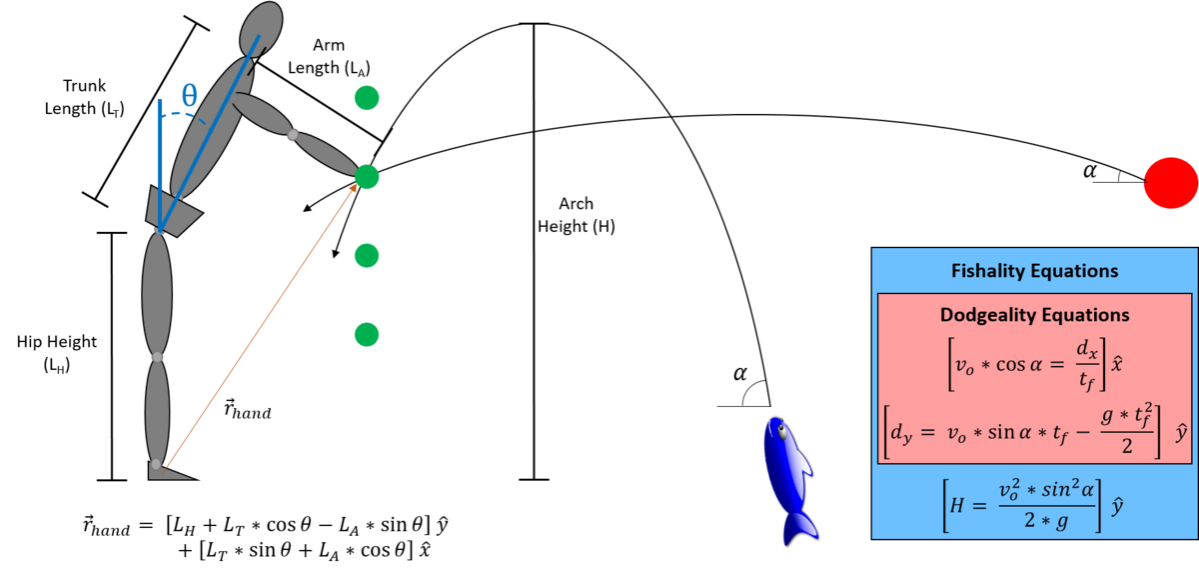
Diagram of the physics equations used in the different VR games. Four target contact locations (shown in green) are computed for each subject based on anthropometrics and a trunk flexion angle (θ) of 15°, 30°, 45°, and 60° and presented as a static target during Reachality. During Dodgeality, targets are launched with a constant initial velocity (vo), and the launch angle (α) is modified to ensure that the launch trajectory intercepts an intended target contact location. During Fishality, the launch velocity and angle are manipulated to ensure that the launch trajectory reaches a target height (H) and intercepts the intended target contact locations. VR: virtual reality.

**Figure 2:**
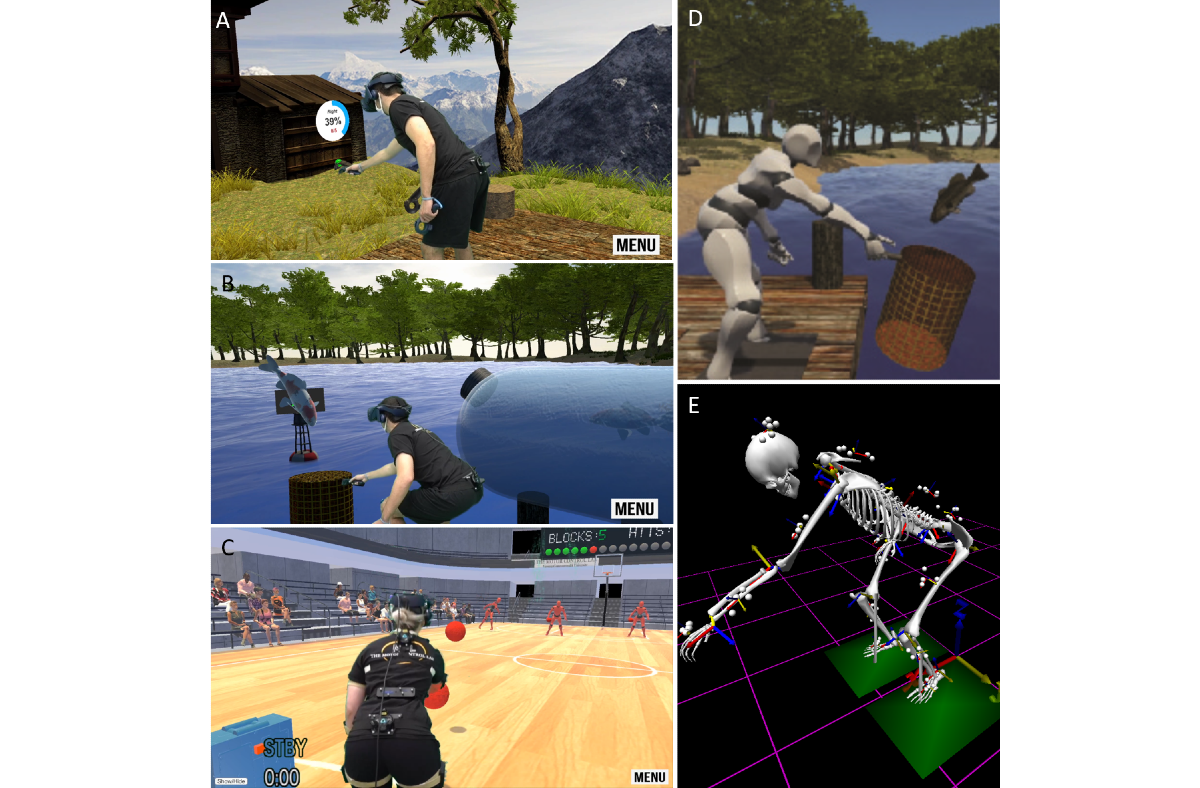
Visual depiction of Reachality (A), Fishality (B), and Dodgeality (C) gameplay, the avatar that participants controlled (D), and the motion analysis data collected during the experiment (E).

A common method to quantify avoidance behavior is through the assessment of motor control during functional tasks that involve kinematic redundancy [12-14]. Our lab uses a standardized reaching task to compare forward reaching across participants with various body anthropometrics [12-14]. This standardized reaching task uses hip height, trunk length, and arm length to compute 4 points in space that would require exactly 15°, 30°, 45°, and 60° of trunk flexion (θ) to reach, if the participant were to move only at the trunk (Figure 1). While these points are computed based on isolated trunk flexion, participants are not instructed as to how they should reach each target. By assessing how an individual chooses to reach each target, this kinematically redundant task is well positioned to identify avoidance behavior (eg, reduced trunk motion and increased motion at the ankle, knee, and hips) [12-14]. While we originally implemented this reaching task with physical targets, we have recently developed a VR version of the task, hereby referred to as “Reachality” (Figure 2A) [15]. In Reachality, participants are immersed in a virtual environment using a head-mounted display, where their body is represented as an avatar and the participants are instructed to reach their hand through targets that appear in front of them (eg, at the 4 aforementioned locations).

Common interventional approaches for patients with LBP and a high fear of movement include graded exposure therapy, wherein patients gradually confront increasingly feared movements, and motor control exercises, wherein existing movement patterns are retrained with a specific focus on restoring trunk control [16-18]. Our group recently developed a VR dodgeball game, hereby referred to as “Dodgeality,” with the goal of creating a gamified intervention that incorporates principles of these classic interventional approaches that are known to be successful [6,19]. In Dodgeality, participants are immersed in a virtual gymnasium and hold a plastic 3D-printed dodgeball, which is tracked and visualized as a dodgeball in the avatar’s hand within the virtual environment. There are 4 opposing players who randomly take turns throwing dodgeballs at the participants, who are instructed to block the balls thrown at them with the ball held in their hand. Each dodgeball is launched at a constant velocity and is modeled as a point-mass projectile with only gravitational forces acting upon it, resulting in a parabolic flight path. Through manipulating only launch angle, we are able to force the dodgeballs’ trajectory to intercept the same 4 locations in space that are presented as targets during Reachality (Figure 1), thereby enticing the participants to reach those locations during gameplay. In a recent phase I clinical trial, our group found that lower intercept locations resulted in increased lumbar flexion excursion in patients with chronic LBP [6]. As avoidance behavior is more prominent when reaching for lower targets [14], the ability to increase the magnitude of trunk flexion needed for gameplay throughout an intervention is essential. Other important findings from the phase I clinical trial were that participants viewed the game as “distracting from their back pain” and “fun to play,” that they were “not worried about hurting their back during gameplay,” and that the game “did not increase their back pain” [6]. Based on the findings from this phase I trial, we believe that Dodgeality could be a useful component of rehabilitation for patients with chronic LBP and a high fear of movement, and we are currently evaluating the efficacy of this intervention in a phase II randomized clinical trial [20].

While our recent findings indicate that we are able to successfully manipulate the amount of trunk flexion needed during gameplay [6], Dodgeality is inherently a fast-paced game that requires quick reactions. In open-ended survey responses, some participants indicated a desire for more practice in the virtual environment at slower speeds [6]. Importantly, a primary principle of graded exposure therapy is to begin with patients’ least feared movement and gradually work toward their most feared movement [16-18]. Patients with LBP and a high fear of movement not only restrict the amount of trunk motion during forward reaching but also the speed at which they flex their trunk [12]. Therefore, to improve our ability to gradually increase mechanical demands of the trunk throughout an intervention, we sought to create a new game that required similar amounts of trunk flexion as Dodgeality but with lower velocity demands. We developed a virtual fish-catching game, hereby referred to as “Fishality,” which is intended to precede Dodgeality in a graded intervention (Figure 2B-2D). In the Fishality virtual environment, participants are standing on a dock overlooking a pond and have a basket in their dominant hand (which is tracked with a controller in the real world). Fish swim toward participants, with an indicator above the level of the water to alert participants to an approaching fish. The fish then jump out of the water toward participants at a high parabolic arc, and participants are instructed to catch the fish in their bucket. This trajectory is intended to give participants more time to react, thereby requiring decreased trunk flexion velocities. Through prescribing the height of the trajectory and resolving the kinematic equations, initial launch angle and launch velocity can be computed to ensure that the fish intercept the same 4 points in space that are used during Reachality and Dodgeality (Figure 1).

The purpose of this study was to compare lumbar kinematics across Reachality, Dodgeality, and Fishality in healthy control participants. Our first hypothesis was that lumbar flexion velocity would be increased during Dodgeality relative to that during Fishality. Our second hypothesis was that the extent of lumbar flexion would not be different between virtual games. While Dodgeality and Fishality are designed such that the trajectories of the launched objects intersect each of the 4 static target locations presented during Reachality, the participants are allowed to intercept the launched objects at any point along their trajectory. Considering that the objects’ trajectories are markedly different between Dodgeality and Fishality, it is possible that observed differences in lumbar kinematics may be explained through differences in the actual interception location (rather than the intended location from which trajectories are initially derived). Therefore, we present the following exploratory third hypothesis: participants would reach further in the forward direction during Fishality and Reachality than during Dodgeality.

## Methods

### Participants and Ethical Considerations

A total of 31 healthy, unimpaired participants (16 males, 15 females; mean age 24.7 years, SD 3.3 years; mean weight 76.05 kg, SD 12.24 kg; mean height 172.5 cm, SD 9.8 cm) completed an informed consent process approved by the Virginia Commonwealth University Human Research Protection Program (HM20014879) and then participated in the present study. Inclusion criteria for the study mandated that all participants be between 18 and 35 years of age. Individuals who were pregnant or had a history of spine or hip surgery, LBP in the previous 6 months, a diagnosis of a neurological, cardiovascular, or musculoskeletal disorder that would interfere with the ability to participate in movement-based VR games, alcohol or drug dependence, significant visual impairment, or a history of motion sickness that would prevent the use of a VR head-mounted display were excluded from participating.

### Gameplay

The order of gameplay was fixed such that Reachality was followed by Fishality and then Dodgeality. During Reachality, participants reached virtual targets that were located in the midsagittal plane at heights that would theoretically elicit 15°, 30°, 45°, and 60° of isolated trunk flexion (Figure 1) [21]. The order of the reaching heights was fixed, starting with the highest target and ending with the lowest target. At each height, participants completed 5 reaches with their right hand, followed by 5 reaches with their left hand. A short rest of approximately 15 seconds was provided between reaches and approximately 2 minutes between heights. For each reach, participants were instructed to stand upright until the virtual target changed color (red to green), after which they were instructed to reach their hand through the virtual target and hold it there for 2 seconds, which was timed and displayed visually using a status bar above the target. After the 2 seconds, the target and status bar disappeared, and the participant was instructed to return to upright stance and wait for the next target to appear.

During Fishality, participants held a controller in their right hand, which was visualized as a basket in the virtual environment, and were instructed to catch fish that jumped out of the water toward them in a path that followed a high parabolic arc. The trajectory of each fish was prescribed such that the fish would intercept the same 4 points in space that were used to theoretically elicit 15°, 30°, 45°, and 60° of isolated trunk flexion; however, the participants were not instructed as to where to catch the fish along its trajectory. Along with catching the fish at different heights, participants were occasionally presented with an ominous audio cue followed by a large shark jumping out of the water toward their head, and they were instructed to duck to avoid the shark.

During Dodgeality, participants held a 3D-printed dodgeball, which was tracked and visualized in the virtual environment, and were instructed to use the ball to block incoming dodgeballs that were thrown at them by 4 opponents. Again, the trajectory of the thrown dodgeball was prescribed to intercepts with the 4 aforementioned points in space, and the participants were free to intercept the dodgeball at any point along its trajectory. Dodgeality also involved occasional ducking, with participants instructed to duck and avoid the incoming dodgeball if they heard a quacking sound, and the color of the incoming ball was black instead of red.

Each participant played Reachality, followed by 1 level of Fishality and then 1 level of Dodgeality. Fishality and Dodgeality each consisted of 2 sets of 15 launched fish (or dodgeballs), with an equal and randomized distribution across the 4 target heights and ducking.

### Instrumentation

Whole body kinematics were collected in 3D at 100 Hz using a 14-camera passive motion capture system (Vero v1.3, Vicon Motion Systems Ltd.) and rigid tracking clusters placed on the head over the thoracic spine, lumbar spine, and pelvis and bilaterally on the feet, shank, thigh, arm, forearm, and hands. Each rigid cluster was 3D printed (Taz 6, LulzBot Inc.), contained 4-7 spherical retroreflective markers (9.5 mm Pearl Markers, B&L Engineering), and was affixed to the body using Velcro straps (Fabrifoam ProWrap, Applied Technology International, Ltd.). The 3D position and orientation of each rigid cluster were recorded at 100 Hz and streamed to a Transmission Control Protocol (TCP) socket port in real time using Vicon Tracker software.

Motion monitor software (MotionMonitor xGEN, Innovative Sports Training Inc.) was used to read the rigid cluster kinematics and kinetic data obtained from 2 embedded force plates (Bertec Inc.). Segment orientations were defined in MotionMonitor xGEN through digitizing anatomic landmarks during quiet stance using a custom 3D-printed stylus pen that contained 5 reflective markers. Segments were then tracked in 6 degrees of freedom during motion, and joint angles were computed between adjacent segments using an Euler angle sequence of rotations in the sagittal, frontal, and transverse planes. All kinematic and kinetic data were recorded for each trial using MotionMonitor xGEN and exported for further analyses.

Along with the motion capture system, participants held a 3D-printed dodgeball, which had a wireless HTC Vive tracker (HTC America Inc.) attached to it, during Dodgeality and held a wireless HTC controller in their right hand during Fishality. The 3D position and orientation of the Vive tracker and controller were tracked using 2 HTC Base Stations, which emit infrared light that is sensed by multiple photodiode detectors on the tracker and the controller to determine orientation. The kinematics of the Vive tracker and controller were also streamed to a TCP socket port in near real time using SteamVR software (Valve Inc.).

The VR environments and games were custom-built using Unity game engine (version 3.9, Unity Technologies). The Unity program read incoming data from Vicon Tracker, MotionMonitor xGEN, and SteamVR from the TCP socket ports and used these data to build and control the participants’ avatar in the virtual environment. Along with reading incoming data, the Unity program also sent data to MotionMonitor xGEN regarding the timing of game events (eg, when the virtual target appeared, cued reaching by changing colors, and was first contacted during Reachality). Participants were immersed in the virtual environment using an HTC Vive-wired, head-mounted display, which presented them with a first-person perspective of their avatar. The head-mounted display had a resolution of 1080 × 1200 per eye, with a refresh rate of 90 Hz and a field of view of 110°.

### Analysis

Joint kinematics exported from MotionMonitor xGEN were further reduced using a custom-built MATLAB program (version 2020a, The MathWorks Inc.). Joint angle time series were smoothed and differentiated using a 41-point, fourth-order Savitzky-Golay filter, which computes polynomial coefficients to fit a least-squares solution to the data [22,23]. Lumbar flexion excursions and peak lumbar flexion velocity were computed for each forward reaching movement. As fishing was played with the right hand, joint kinematics were assessed on the right side and only right-handed reaching trials were analyzed. Joint excursion and velocity were computed between the time when each movement began (eg, target appeared in Reachality, opponent began winding up in Dodgeality, or fish began swimming toward the participant in Fishality) and 200 milliseconds after the participant contacted the target (or fish/dodgeball). Trials where the targets were not successfully intercepted (ie, the fish was not caught in the basket) were included in the analyses as long as the participant did react to the launch and attempted to intercept it. Each trial was visually reviewed by a member of the study team, and if there was an apparent lack of lumbar motion in response to a launched target, that trial was excluded from analysis. Along with lumbar excursion and velocity, hand location at target contact was computed relative to the midpoint of the participants’ feet. Outcomes were computed for each movement and then averaged across reaching height for each game.

### Statistical Analysis

Data were tested for normality using Shapiro-Wilk tests before separate 2-way repeated measures analyses of variance were performed for each outcome measure, with game (Reachality, Fishality, and Dodgeality) and height (target location for 15°, 30°, 45°, and 60° of trunk flexion) as within-subject variables. Greenhouse-Geisser corrections were applied when the assumption of sphericity was not met. Effect sizes (via partial Eta-squared values) were computed for each analysis of variance model, with values greater than 0.25 indicating a moderate effect and values greater than 0.64 indicating a strong effect [24]. Post-hoc analyses were performed using the method of least significant differences for significant main effects, and interactions were analyzed using a simple effects model. Significance was set at an α level of .05, and all statistical analyses were performed using SPSS (version 27, IBM Inc.).

## Results

**Figure 3:**
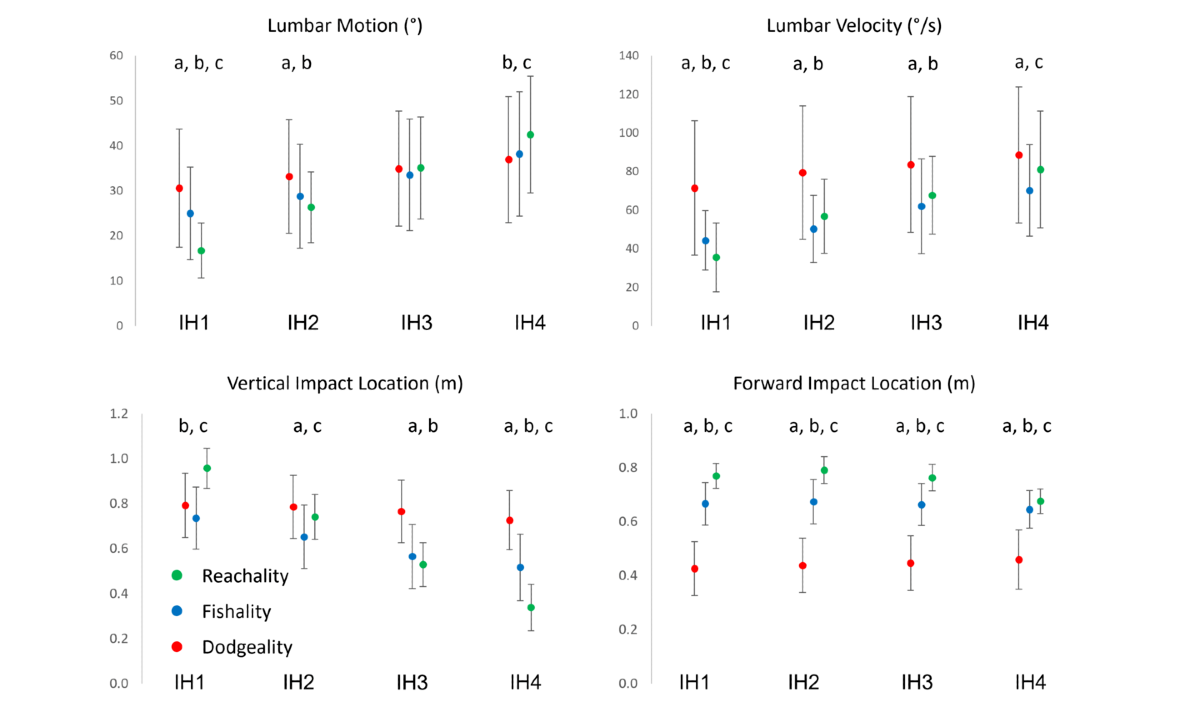
Study outcomes compared across intended impact heights (IH1–IH4) and virtual reality games (Reachality, Fishality, and Dodgeality). Error bars represent 1 standard deviation. a: significant difference between Dodgeality and Fishality; b: significant difference between Dodgeality and Reachality; c: significant difference between Fishality and Reachality.

The raw data and results for the repeated measures analysis of variance are presented in Table 1. There was an interaction between game and impact height for each outcome (Figure 3, all *P*<.001). Therefore, post-hoc simple effects models (ie, 1-way analysis of variance) were performed to compare the 3 games across each impact height.

**Table 1:** Outcome measures compared across games and impact heights.

Outcome measures	Game	Impact height	Interaction between game and impact height
Lumbar motion (°)	F(2,48)=2.739 *P*=.08 *η*^2^=0.102	F(1.4,33.9)=110.41 *P*<.001 *η*^2^=0.821	F(2.9,69.7)=22.092 *P*<.001 *η*^2^=0.479
Lumbar velocity (°/s)	F(2,48)=17.002 *P*<.001 *η*^2^=0.415	F(1.4,34.2)=108.151 *P*<.001 *η*^2^=0.818	F(3.4,82.6)=9.366 *P*<.001 *η*^2^=0.281
Anterior-posterior impact location (m)	F(1.4,32.4)=136.48 *P*<.001 *η*^2^=0.85	F(3,72)=29.704 *P*<.001 *η*^2^=0.553	F(4.1,97.9)=12.188 *P*<.001 *η*^2^=0.561
Vertical impact location (m)	F(1.6,37.8)=16.653 *P*<.001 *η*^2^=0.41	F(2.1,51.1)=493.625 *P*<.001 *η*^2^=0.954	F(3.8,91.0)=150.701 *P*<.001 *η*^2^=0.863

### Lumbar Excursions

On examination of the effects of game type on lumbar excursions, the effects were found to be different at each intended impact height. Specifically, at intended impact height 1, lumbar flexion excursion was greater during Dodgeality than during Fishality and Reachality and greater during Fishality than during Reachality. At intended impact height 2, lumbar flexion excursion was greater during Dodgeality than during Fishality and Reachality. There were no significant differences between the games at intended impact height 3, but at intended impact height 4, lumbar flexion excursion was greater during Reachality than during Dodgeality and Fishality.

### Lumbar Flexion Velocity

On examination of the effects of game type on lumbar flexion velocity, the effects were found to be different at each intended impact height. Specifically, at intended impact height 1, lumbar flexion velocity was greater during Dodgeality than during Fishality and Reachality and greater during Fishality than during Reachality. At intended impact heights 2 and 3, lumbar flexion velocity was greater during Dodgeality than during Fishality and Reachality. Finally, at intended impact height 4, lumbar flexion velocity was greater during Dodgeality and Reachality than during Fishality.

### Impact Location

The differences between games for actual impact location in the vertical direction varied across intended impact heights. At intended impact height 1, actual impact location was lower during Dodgeality and Fishality than during Reachality. At intended impact height 2, actual impact location was lower during Fishality than during both Dodgeality and Reachality. At intended impact height 3, actual impact location was lower during Fishality and Reachality than during Dodgeality. Finally, at intended impact height 4, actual impact location was lower during Reachality than during Dodgeality and Fishality and lower during Fishality than during Dodgeality. At each intended impact height, the actual impact location in the anterior-posterior direction was greater during Reachality than during Dodgeality and Fishality and greater during Fishality than during Dodgeality. 

## Discussion

Gamified movement-based intervention is a promising approach for rehabilitation in patients with LBP and a high fear of movement. Our group recently developed Dodgeality, a virtual dodgeball game where patients are encouraged to bend forward to block incoming balls thrown at them by opposing players [6]. While we are able to influence the amount of trunk flexion needed for successful gameplay by modifying the intended impact location of launched balls, Dodgeality is inherently a fast-paced game with large movement velocity demands. We sought to supplement our VR game suite by developing a game to encourage patients to flex their trunk at a slower speed. We therefore developed Fishality, a novel VR game where fish jump out of a body of water with a high parabolic arc, and the patients have to bend forward to catch the fish before they land back in the water.

This study sought to compare movement biomechanics across Dodgeality, Fishality, and a standardized virtual reaching task (Reachality). Our first hypothesis was supported, as lumbar flexion velocity was greater during Dodgeality than during Fishality. While flexion velocity during Fishality was less than that during Dodgeality at each intended impact height, differences were greater for higher impact heights (requiring less motion). Specifically, lumbar flexion velocity reduced by 38% at intended impact height 1 (Dodgeality: mean 71.5°, SD 35.8°; Fishality: mean 44.3°, SD 15.4°) and 21% at intended impact height 4 (Dodgeality: mean 88.7°, SD 35.3°; Fishality: mean 70.2°, SD 23.7°). Our second hypothesis was not supported, as lumbar motion was different between games. Specifically, Fishality resulted in 13%-18% less lumbar motion relative to Dodgeality for higher targets, resulting in magnitudes of movement that were more similar to those for Reachality. It is unclear why lumbar flexion was increased for higher targets during Dodgeality; however, it is likely that participants began moving downward before they identified the target of the incoming ball because of the fast speeds at which the dodgeballs were launched. This finding suggests that Fishality is better than Dodgeality for manipulating trunk flexion during gameplay. As the magnitude of lumbar flexion and lumbar flexion velocity across VR games and impact heights were comparable between this study and prior research conducted in a real-world environment [12,21], the findings of this study were likely not due to the VR environment itself. Additional evidence for this notion comes from a prior study that found limited differences in lumbar motion and velocity when reaching tasks were compared between a virtual environment and real-world setting [15]. Because a major goal of graded intervention is to increase movement demands throughout the course of an intervention [16-18], the ability to modulate lumbar motion and velocity is essential. Hence, the results of this study are encouraging when considering the development of an individualized, graded intervention program for patients with LBP who have low physical activity and a high fear of movement.

Another important finding from this study was that participants did not reach as far forward when playing Dodgeality and Fishality relative to when making contact with the targets during Reachality. This finding intuitively makes sense, as projectiles (dodgeball and fish) can be intercepted at any point along their trajectory for successful gameplay during Dodgeality and Fishality (compared to static target positions used in Reachality). Reach distance was increased during Fishality compared to that during Dodgeality, which is also to be expected given the different requirements of the 2 games. Specifically, incoming dodgeballs have a flat trajectory that will contact participants’ bodies if not blocked, whereas incoming fish have a high parabolic trajectory and will land in the water between the participant and intended intercept target if not caught. Based on these findings, feature modifications to our VR games, such as interception boundaries, could be introduced to ensure greater forward movement during gameplay, which would improve our ability to specifically target lumbar flexion appropriately across all target heights.

This study had limitations that should be considered when interpreting the findings. First, as our sample consisted of young healthy participants, these findings should be repeated in a cohort of participants with a broad range of ages and spine impairments to determine the robustness of the findings. However, for this study, we intentionally included healthy participants without impairment to ensure that the task demands aligned with how we had developed the VR games. A second potential limitation is that the gameplay order was not randomized, which could have introduced an ordering effect into our data. However, as the games are designed to be used in an ordered fashion for interventional purposes, we wished to investigate movement behaviors within this context.

In conclusion, the present study sought to compare a virtual dodgeball game, a newly developed virtual fish-catching game, and a virtual reaching task in a healthy sample. We found that lumbar flexion velocities were reduced in Fishality compared to those in Dodgeality and resembled velocity demands more similar to those in a self-paced reaching task (ie, Reachality). These findings are encouraging for future research aimed at developing individualized, graded VR interventions for patients with LBP and a high fear of movement.

## Abbreviations

LBP: low back pain
TCP: Transmission Control Protocol
VR: virtual reality
